# Aged Gut Microbiome Induces Metabolic Impairment and Hallmarks of Vascular and Intestinal Aging in Young Mice

**DOI:** 10.3390/antiox13101250

**Published:** 2024-10-17

**Authors:** Chak-Kwong Cheng, Lianwei Ye, Yuanyuan Zuo, Yaling Wang, Li Wang, Fuyong Li, Sheng Chen, Yu Huang

**Affiliations:** 1 Department of Biomedical Sciences, City University of Hong Kong, Hong Kong SAR, China; li.wang@cityu.edu.hk; 2 Department of Infectious Diseases and Public Health, Jockey Club College of Veterinary Medicine and Life Sciences, City University of Hong Kong, Hong Kong SAR, China; lianweiye2-c@my.cityu.edu.hk (L.Y.); fuyongli@cityu.edu.hk (F.L.); 3 School of Biomedical Sciences, The Chinese University of Hong Kong, Hong Kong SAR, China; zyyeccom3@gmail.com; 4 Department of Food Science and Nutrition, The Hong Kong Polytechnic University, Hong Kong SAR, China; gstwyla@polyu.edu.hk (Y.W.); sheng.chen@polyu.edu.hk (S.C.)

**Keywords:** AMPK, aging, dysbiosis, endothelial cell, microbiome, telomeres

## Abstract

Aging, an independent risk factor for cardiometabolic diseases, refers to a progressive deterioration in physiological function, characterized by 12 established hallmarks. Vascular aging is driven by endothelial dysfunction, telomere dysfunction, oxidative stress, and vascular inflammation. This study investigated whether aged gut microbiome promotes vascular aging and metabolic impairment. Fecal microbiome transfer (FMT) was conducted from aged (>75 weeks old) to young C57BL/6 mice (8 weeks old) for 6 weeks. Wire myography was used to evaluate endothelial function in aortas and mesenteric arteries. ROS levels were measured by dihydroethidium (DHE) staining and lucigenin-enhanced chemiluminescence. Vascular and intestinal telomere function, in terms of relative telomere length, telomerase reverse transcriptase expression and telomerase activity, were measured. Systemic inflammation, endotoxemia and intestinal integrity of mice were assessed. Gut microbiome profiles were studied by 16S rRNA sequencing. Some middle-aged mice (40–42 weeks old) were subjected to chronic metformin treatment and exercise training for 4 weeks to evaluate their anti-aging benefits. Six-week FMT impaired glucose homeostasis and caused vascular dysfunction in aortas and mesenteric arteries in young mice. FMT triggered vascular inflammation and oxidative stress, along with declined telomerase activity and shorter telomere length in aortas. Additionally, FMT impaired intestinal integrity, and triggered AMPK inactivation and telomere dysfunction in intestines, potentially attributed to the altered gut microbial profiles. Metformin treatment and moderate exercise improved integrity, AMPK activation and telomere function in mouse intestines. Our data highlight aged microbiome as a mechanism that accelerates intestinal and vascular aging, suggesting the gut-vascular connection as a potential intervention target against cardiovascular aging and complications.

## 1. Introduction

Aging is generally considered as a progressive decline of physiological integrity, accompanied by increasing risks of disease and mortality [1]. Aging significantly increases the risks for cardiovascular diseases (CVDs) and diabetes mellitus. Until 2023, aging is suggested to be characterized by 12 primary, antagonistic and integrative hallmarks: genomic instability, telomere attrition, deregulated nutrient-sensing, loss of proteostasis, epigenetic alterations, chronic inflammation, cellular senescence, altered intercellular communication, mitochondrial dysfunction, disabled macroautophagy, stem cell exhaustion, and dysbiosis [2]. Importantly, a growing number of studies aim at expanding the sophisticated network among these hallmarks in different age-associated diseases.

The aging vasculature is associated with endothelial dysfunction, arterial stiffness, increased oxidative stress and chronic inflammation [3], contributory to higher risks of cardiovascular diseases, such as heart failure and coronary artery disease. ‘*Dysbiosis*’ is considered as a new integrative hallmark of aging. During aging, the microbial composition in intestine varies that the homeostatic relationship between the host and gut microbiome deteriorates, contributory to altered immune and inflammatory responses in the elderly [2]. Age-associated dysbiosis also increases the risks of various diseases, particularly cancers, CVDs, and diabetes, potentially through shift in intestinal microbial composition from providing benefits to causing chronic inflammation, and through alterations in the production of gut-derived substances to influence host nutrient-sensing pathways [4]. However, the comprehensive mechanism of how age-related dysbiosis promotes other recognized hallmarks of aging remains elusive.

Of note, the vasculature serves as the first-line barrier vulnerable to the changes in gut microbiome due to close proximity between the intestine and blood circulation [5]. Besides, the increased intestinal permeability, known as ‘leaky gut’, during aging further aggravates the vulnerability of vasculature towards dysbiosis [6]. However, whether age-associated dysbiosis promotes host premature aging remains unclear. Previous studies showed that gut microbiome suppression by antibiotics ameliorates endothelial dysfunction in aged mice [7], and age-associated hyperproduction of harmful gut-derived metabolite (e.g., trimethylamine-N-oxide) drives endothelial dysfunction [8]. It is therefore reasonable to hypothesize that age-associated dysbiosis shall promote vascular aging by triggering aging hallmarks in the vasculature, such as telomere attrition, endothelial dysfunction, and vascular inflammation and oxidative stress.

Additionally, most previous studies mainly focused on whether certain interventions could retard or reverse aging in aged individuals and animals. In other words, it is also important to investigate whether certain interventions would accelerate aging for future prevention. Therefore, in this study, we performed fecal microbiome transfer (FMT) from aged to young mice to (i) study whether age-associated dysbiosis causes premature vascular and intestinal dysfunction; (ii) unveil whether such transfer changes the metabolic profiles; (iii) uncover microbiome alterations and underlying mechanism that are potentially critical; and (iv) identify potential interventions that might partially reverse age-related harmful effects. Our findings shall highlight that the gut-vascular connection can be a potential intervention target for alternative therapies against age-related cardiometabolic complications.

## 2. Materials and Methods

### 2.1. Ethics Approval Statement and Animal Experimentation

All animal experiments were performed in compliance with the Guide for the Care and Use of Laboratory Animals (National Institutes of Health), the ARRIVE guidelines, and the ethical guidelines established by the Animal Research Ethics Sub-Committee (ethical approval number: AN-STA-00000132), City University of Hong Kong (CityU). Specific pathogen-free (SPF) male young (8 weeks old), middle-aged (40–42 weeks old), and aged (>75 weeks old) C57BL/6 mice were supplied by Laboratory Animal Research Unit (LARU) of CityU. The mice were maintained in individually ventilated cages under a 12/12 h light/dark cycle, with free access to water and laboratory food. The temperature (22 ± 2 °C) and humidity (50% ± 10%) were maintained constant in an SPF animal facility. Animal randomization was performed prior to experiments.

### 2.2. Metformin Administration

Some middle-aged C57/BL6 mice (40–42 weeks old) were orally administered with the anti-diabetic drug metformin (100 mg/kg; Sigma-Aldrich, St. Louis, MO, USA), an 5′-adenosine monophosphate-activated protein kinase (AMPK) activator, for 4 weeks. This metformin dosage is considered safe for in vivo animal experimentation as suggested by previous studies [9]. Some middle-aged mice were orally gavaged with compound C (20 mg/kg; Sigma-Aldrich), an AMPK inhibitor, once daily for 2 weeks.

### 2.3. Fecal Microbiome Transfer (FMT)

**Figure 1:**
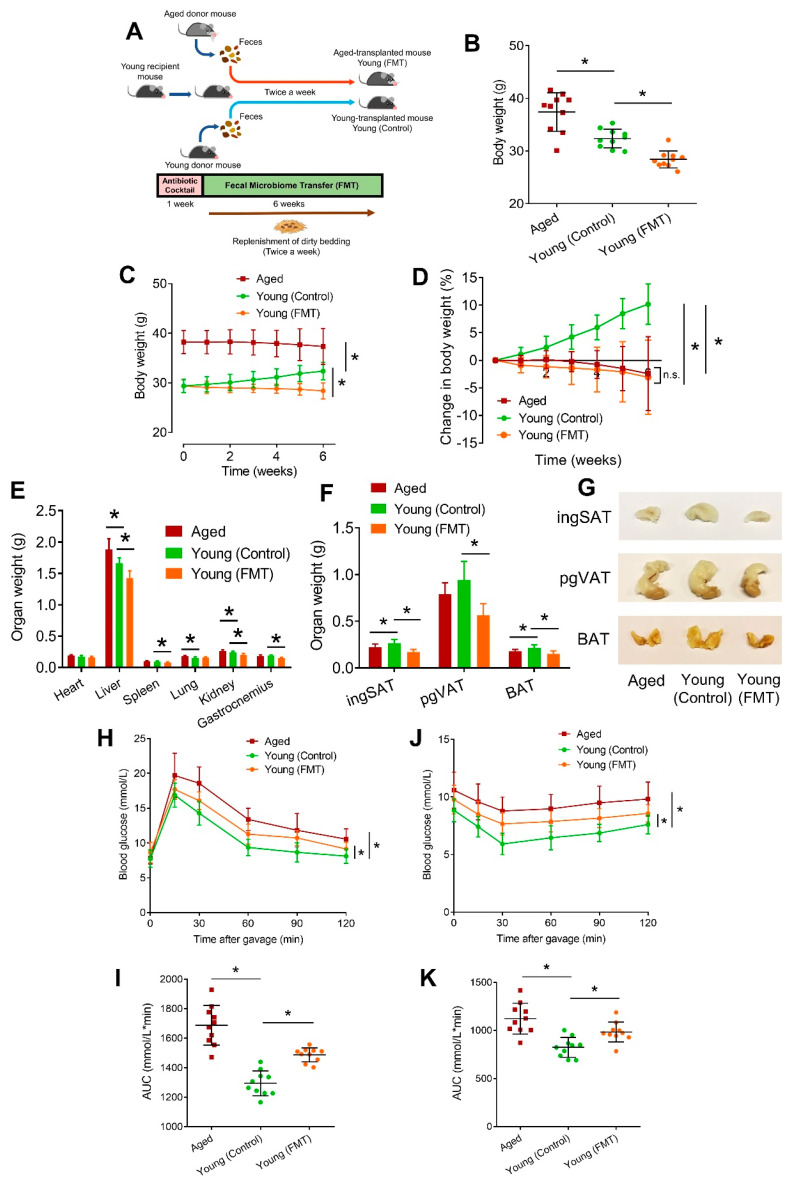
Effects of aged-to-young FMT on body parameters. (**A**) Schematic overview on FMT protocol from aged and young donor mice to young recipient mice. (**B**) Body weights of aged donor mice (Aged), young-transplanted (Young (Control)) and aged-transplanted young mice (Young (FMT)) after 6-week FMT protocol. (**C**) Body weight changes and (**D**) percentage changes in body weights of mice in (**B**) during the 6-week FMT. (**E**) Weights of indicated organs of mice in (**B**) postmortem after the 6-week FMT. (**F**) Weights of inguinal subcutaneous adipose tissue (ingSAT), perigonadal visceral adipose tissue (pgVAT) and brown adipose tissue (BAT) of mice in (**B**). (**G**) Gross appearance of adipose tissues of mice in (**B**). (**H**) Glucose tolerance test (GTT) on mice in (**B**) at week 6 of FMT, and (**I**) corresponding area under curve (AUC) analysis of glucose over time. (**J**) Insulin tolerance test (ITT) of mice in (**B**) at week 6 of FMT, and (**K**) corresponding AUC analysis of glucose over time. *N* = 10 per group. Data are mean ± SD. * *p* < 0.05; Brown-Forsythe and Welch ANOVA and Dunnett T3 test.

Before FMT, young recipient C57BL/6 mice (both control and FMT groups), but not the donor C57BL/6 mice, were subjected to a 1-week treatment of antibiotic cocktail-containing drinking water (ampicillin: 1 g/L; 0.5 g/L; metronidazole: 1 g/L; neomycin: 0.5 g/L; vancomycin: 0.5 g/L) [10]. Antibiotic pretreatment was shortened to 1 week to avoid any adverse effects due to prolonged antibiotic exposure. Starting from the first day after antibiotic administration, FMT was performed by oral gavage of microbiome suspension (150 μL) from either aged or age-matched young donor mice to young recipient mice semiweekly for 6 weeks (9 mouse days~1 human year; 42 mouse days ~4.667 human years; 8 mouse weeks ~6.222 human years; 40 mouse weeks~31.111 human years; 75 mouse weeks ~58.333 human years) [11] (Figure 1A). Fecal pellets pooled from 8–10 donor mice were freshly collected for the preparation of microbiome suspension. The weighed fecal pellets (300 mg) were resuspended by 1-min vortexing in 1 mL sterile PBS. Only supernatant was collected for subsequent FMT, where any insolubilized material was removed by centrifugation (500× *g*, 5 min). The cages of Young (FMT) mice and Young (Control) mice were respectively replenished with dirty bedding and fresh stools from aged or aged-matched young donor mice semiweekly [12]. Body weights and the food intake amount of different mouse groups were documented weekly over the FMT period. After the 6-week FMT, the weights of indicated organs and adipose tissues were recorded after CO_2_ euthanasia of mice.

### 2.4. Exercise Protocol

Some middle-aged mice (40–42 weeks old) were trained on a motorized running treadmill. The mice were acclimatized for 1 week, during which exercise intensity and duration gradually elevated. The treadmill velocity was gradually increased from 5 m/min to 8 m/min (total time: 30 min; total distance: 240 m), where the exercise intensity was well tolerated by mice as shown in a previous report [13]. Ater 1-week acclimatization, treadmill exercise was performed for 4 consecutive weeks (6 days/week; total: 5 weeks). For control group, mice were kept sedentary on a static treadmill over the same time period (30 min) as those of exercise-trained mice. Compound C (20 mg/kg) was orally gavaged in some middle-aged mice for 2 weeks (Figure 6A).

### 2.5. Blood Glucose Measurement

At 3rd and 6th weeks of FMT, blood glucose levels were measured. Mice were fasted by 16 h and 2 h for oral glucose tolerance test (OGTT) and insulin tolerance test (ITT), respectively. After fasting, OGTT was then conducted after oral gavage of glucose (1.2 g/kg), whereas ITT was conducted by intraperitoneal insulin injection (1 unit/kg) [14]. At specified time points (0, 15, 30, 60, 90 and 120 min), blood glucose levels were assessed in blood from mouse tail veins.

### 2.6. Blood Pressure Measurement

Mouse blood pressure was measured via tail-cuff technique non-invasively by the CODA^®^ High Throughput System (Kent Scientific, Torrington, WY, USA). Briefly, mice were acclimated to restrainers, which were put on a warming board for steady body temperature (37 °C) maintenance in a quiet environment. Mean arterial pressure was calculated from an average of 15 recordings.

### 2.7. Serum Lipid Profile

Mouse blood was obtained through celiac vein after sacrifice. Serum was collected by a 10-min centrifugation (3000 rpm, room temperature). Lipid profile on serum total cholesterol (TC), high-density lipoprotein (HDL) cholesterol, and triglycerides (TG) was determined by a commercial assay kit (Stanbio, Boerne, USA). HDL isolation from whole serum was accomplished by adding Stanbio HDL precipitating reagent (1:10), followed by centrifugation (1000× *g*, 10 min). Levels of TC, HDL, and TG were later measured by a plate reader (500 nm; Bio-Rad, Hercules, USA) based on the manufacturer’s protocol. According to the formula: non-HDL cholesterol = TC-HDL-(TG/5), non-HDL cholesterol levels can be determined [15].

### 2.8. Functional Assay by Wire Myography

After mouse CO_2_ euthanasia, mouse thoracic aortas and second-order mesenteric arteries were isolated with careful removal of surrounding connective tissues in sterile PBS. The arteries were immediately segmented into ~2 mm circular segments in oxygenated ice-cold Krebs solution containing (in mmol/L): 11 D-glucose, 119 NaCl, 25 NaHCO_3_, 4.7 KCl, 1.2 KH_2_PO_4_, 1 MgCl_2_, and 2.5 CaCl_2_. The segments were then mounted on individual channels of Wire Myograph System (Danish Myo Technology, Hinnerup, Denmark) for vascular tone determination. Before the assay, each segment was factitiously stretched to a baseline tension (aorta: 3 mN; mesenteric artery: 2 mN) and underwent a 1-hr equilibrium in oxygenated Krebs solution (95% O_2_, 5% CO_2_) at 37 °C. The segments were firstly pre-contracted by KCl (60 mmol/L), followed by Krebs rinsing for three times. Phenylephrine (Phe; 3 μmol/L; Sigma-Aldrich) was added to induce vascular contraction. Then acetylcholine (ACh; Sigma-Aldrich) was cumulatively added (3 nmol/L–10 μmol/L) to trigger endothelium-dependent relaxations (EDRs). Some mesenteric arteries were subjected to a 30-min pre-incubation with the NOS inhibitor N^G^-nitro-L-arginine methyl ester (L-NAME; 100 μmol/L; Sigma-Aldrich) before EDR induction. Sodium nitroprusside (SNP; Sigma-Aldrich) was cumulatively added (1 nmol/L–10 μmol/L) to assess endothelium-independent relaxations. A PowerLab LabChart 8.0 system (AD Instruments, Sydney, Australia) was used to document tensional changes of arteries [16].

### 2.9. ROS Detection by Dihydroethidium (DHE) Staining

DHE staining was applied for ROS detection in mouse aortas (en face endothelium). Aortas was freshly dissected into aortic rings (~2 mm) for subsequent incubation in normal physiological saline solution (NPSS) containing (in mmol/L): 10 glucose, 140 NaCl, 5 HEPES, 5 KCl, 1 MgCl_2_, and 1 CaCl_2_) at pH 7.4, supplemented with DHE (5 μmol/L; Invitrogen, Waltham, MA, USA). After that, the aortic rings were washed in NPSS for three times, followed by a longitudinal cut for en face endothelium exposure. Fluorescent signals of DHE (excitation: 515 nm; emission: 585 nm) were assessed by the Olympus FluoView^TM^ FV1000 system (Olympus, Shinjuku City, Japan). The elastin autofluorescent signals (excitation: 488 nm; emission: 520–535 nm) were also assessed. The DHE signals were displayed in real numbers.

### 2.10. Lucigenin-Amplified Chemiluminescence Assay

The generation of superoxide anions from aortic and intestinal tissues were assayed by lucigenin-amplified chemiluminescence. The mouse aortic and intestinal tissues were freshly collected for a 45 min-incubation at 37 °C in Krebs-HEPES solution containing (in mmol/L): 11 glucose, 99 NaCl, 25 NaHCO_3_, 20 Na-HEPES, 4.7 KCl, 1 KH_2_PO_4_, 1.2 MgSO_4_, and 2.5 CaCl_2_, supplemented with β-NADPH (0.1 mmol/L; Sigma-Aldrich) and diethyldithiocarbamic acid (DDC; 1 mmol/L; Sigma-Aldrich). The incubated tissues were subsequently put into vials of Krebs-HEPES solution, supplemented with 10 μmol/L lucigenin (Sigma-Aldrich). In 1-min intervals, measurements were repeatedly noted by a GloMax^®^ 20/20 Luminometer (Promega, Madison, WI, USA) for 10 min. The amount of superoxide anion generated was displayed in real numbers in relative light units (RLU)/dry tissues (mg) [17].

### 2.11. Western Blotting

Protein extracts from aortic and intestinal tissues were obtained by homogenizing the tissues in RIPA buffer, containing protease inhibitor cocktail (Sigma-Aldrich) and phosphatase inhibitor (Roche, Basel, Switzerland), on ice. A commercially available BCA protein assay kit (Pierce Biotechnology, Waltham, MA, USA) was used to measure protein contents. The protein samples were then mixed with 5× loading buffer (5% β-mercaptoethanol), followed by a 5-min denaturation at 95 °C. Equal protein amount (20 μg) was loaded and resolved by a SDS-polyacrylamide gel (10%) along with a protein marker (Thermo Scientific, Waltham, MA, USA). The resolved proteins were subsequently transferred to a PVDF membrane (Millipore, Burlington, VT, USA). The blots were blocked for 30 min in TBS, supplemented with 3% BSA and 0.05% Tween-20. After blocking, the blots were incubated overnight at 4 °C with primary antibodies: anti-β-tubulin (1:1000; HC101; Transgen Biotech, Beijing, China), anti-AMPK (1:1000; #2532; Cell Signaling Technology (CST), Danvers, MA, USA), anti-phospho-AMPK at Thr172 (1:1000; #2535; CST), anti-eNOS (1:1000; #610297; BD Transduction Laboratory, Franklin Lakes, NJ, USA), and anti-phospho-eNOS at Ser1177 (1:1000; #9571S; CST). The blots were then incubated at room temperature for 2 h with horseradish peroxidase-conjugated secondary antibodies (CST). ChemiDoc^TM^ Imaging System (Bio-Rad, Hercules, CA, USA) was applied to visualize protein bands.

### 2.12. Nitrite Assay

After ACh pretreatment (10 μmol/L) in mouse aortas at 37 °C for 10 min to induce generation of nitric oxide (NO), the aortas were subsequently incubated with nitrate reductase for nitrate-to-nitrite reduction. After homogenizing the aortas, the nitrite levels of the collected supernatants were determined by a commercially available colorimetric assay kit (Molecular Probes, Eugene, OR, USA), by measuring absorbance at 548 nm. The measured absorbance was compared to a standard nitrite curve for obtaining nitrite values.

### 2.13. Quantitative Real-Time PCR

Total RNA extraction from tissues were performed by using TRIzol reagent (Invitrogen). For mRNA quantification, iScript^TM^ cDNA synthesis kit ((Bio-Rad, Hercules, CA, USA) was used to synthesize cDNAs. RT-PCR was conducted by using SYBR Premix ExTaq (TaKaRa, Kusatsu, Japan) in ABI ViiA7 system. Primer pairs of the current study were included in Table S1. Gapdh was chosen as the endogenous control.

### 2.14. ELISA

Mouse blood was collected via celiac vein after sacrifice, followed by serum collection by centrifugation (3000 rpm, 10 min) at room temperature. Circulating levels of inflammatory cytokines, including tumor necrosis factor α (TNFα) and interleukin (IL)-6, were quantified by ELISA kits (Invitrogen) following standard kit protocol. Circulating level of glucagon-like peptide-1 (GLP-1) was measured by another ELISA kit (Sigma-Aldrich) according to the standard kit protocol.

### 2.15. Telomerase Activity Quantification

Telomerase activity in tissues was quantified by a RT-PCR approach with the aid of TRAPeze^®^ RT Telomerase Detection Kit (Merck, Darmstadt, Germany). In brief, telomerase extraction was performed by CHAPS lysis buffer (200 μL/50 mg tissue)-mediated tissue lysis in homogenizer. Bradford method was adopted to measure protein concentrations for subsequent normalization to 500 ng/μL. To prepare the master mix, 5× TRAPEZE^®^ RT reaction mix containing Amplifluor^®^ primers, Taq Polymerase (5 units/μL; Thermo Scientific) and nuclease-free water were mixed. The master mix was then aliquoted to a 96-well plate. Sample randomization and duplicate measurement were adopted. Standard curves on TSR8 control (1:10 serial dilutions; 0.4–0.0004 attomoles), negative control and positive control were then examined. The values of telomerase activity were obtained from the standard curve on TSR8 control [18].

### 2.16. Measurment of Telomere Length

As previously documented, relative telomere lengths in mouse tissues were calculated by using a RT-PCR approach [19]. In brief, genomic DNA extraction was accomplished by using the commercially available purification kit (Thermo Scientific). Specific primer pairs for acidic ribosomal phosphoprotein (36B4) gene and telomere were used for subsequent RT-PCR in ABI ViiA7 system (Table S1). 36B4 is a single copy reference gene for endogenous control. The relative telomere length of tissues was determined based on ΔCT value calculation.

### 2.17. Detection of Endotoxemia and Intestinal Barrier Dysfunction

To detect the presence of endotoxemia, levels of lipopolysaccharide (LPS) in serum and stools, and LPS-binding protein (LBP) in serum were quantified. Serum endotoxin levels were measured by the LAL Chromogenic Endotoxin Quantitation kit (Pierce, Waltham, MA, USA). Serum samples were firstly subjected to 1:50–1:100 dilutions in sterile conditions, and were inactivated at 70 °C for 15 min. Meanwhile, fecal endotoxin levels were measured by the same kit with an amended protocol. In brief, mouse fecal pellets were subjected to a 60-min sonication in 10 mL sterile PBS. The sonicated fecal samples were subjected to centrifugation (400× *g*, 15 min) and filtration through 0.22 μm filters. The filtered samples underwent a 1:1000 dilution in sterile water, and were inactivated at 70 °C for 15 min [20]. Fecal endotoxin quantification was then performed following the manufacturer’s protocol. Meanwhile, concentrations of LBP and intestinal fatty-acid binding protein (I-FABP), the surrogate biomarkers of increased epithelial barrier permeability [21], in mouse serum were respectively quantified by ELISA kits supplied by Invitrogen and MyBioSource (San Diego, CA, USA) based on the standard kit protocol.

### 2.18. DNA Extraction from Fecal Samples

Fecal pellets were collected from mice before and after the FMT protocol. Fecal DNA extraction was performed with the aid of the NucleoSpin^®^ DNA Stool kit (Macherey-Nagel, Düren, Germany). Briefly, fecal pellets (180–200 mg) were subjected to a 10-min vortexing at maximal speed for bead-based homogenization. Total bacterial DNA was retained on the silica membrane, where impurity removal was conducted by applying NucleoSpin^®^ Inhibitor Removal Column to avoid interference on downstream procedures. Later, 80 μL Elution Buffer was used to elute purified DNA, followed by NanoDrop quantification (260 nm).

### 2.19. 16S rRNA Sequencing

Qubit^®^ dsDNA HS Assay Kit was used to monitor DNA concentrations. Preparation of sequencing libraries and Illumina sequencing were performed by Genewiz, Inc. (Azenta Life Sciences, Burlington, NJ, USA). A MetaVX Library Preparation Kit (Genewiz, Inc., South Plainfield, NJ, USA) was applied for the construction of the sequencing library. Briefly, DNA (20–30 ng) was used to produce amplicons targeting hypervariable regions (V3 and V4) of 16S rRNA gene (forward primer: CCTACGGRRBGCASCAGKVRVGAAT; reverse primer: GGACTACVSGGGTATCTAAT). Magnetic bead-based purification of PCR products was performed, where an Infinite 200 Pro microplate reader (Tecan, Männedorf, Switzerland) was applied to detect concentrations. An Illumina Miseq/Novaseq Platform (Illumina, San Diego, CA, USA) was applied for next generation sequencing. Following standard instructions, 250/300 paired-end sequencing with dual reads and automated cluster generation were conducted.

### 2.20. 16S rRNA Sequence Data Analysis

The raw sequencing data were initially processed through splicing, filtering, and de-chimerization steps for obtaining clean data, and for enhancing the accuracy and reliability. Subsequently, sequences sharing a similarity of at least 97% were grouped into the same operational taxonomic unit (OTU). Alpha diversity within each sample and sequencing depth among groups were assessed by calculating rarefaction curves, species accumulation curves, Chao1 index, and ACE index. Statistical analysis on community structure at various taxonomic levels (phylum, class, order, family, genus, and species) was performed using the taxonomic information. To examine the similarity in bacterial community structures among groups, Beta diversity was studied and was visualized through Non-Metric Multi-Dimensional Scaling Analysis (NMDS) and Principal Component Analysis (PCA). Differential abundance (DA) analyses at the species level were performed using the limma package and the voom function for transforming normalized counts into log2-counts-per-million (logCPM). Microbial diversity analysis and taxa plots were generated using the R software (version 4.3.1) along with the qiime2, vegan and ggplot2 packages.

### 2.21. Statistical Analysis

Data are means ± SD. Statistical analyses were conducted by GraphPad Prism software (Version 8.0). For two-tailed comparison between two groups, statistical significance was assessed by unpaired *t*-test and nonparametric Mann-Whitney test. For multiple-group comparison, statistical significance was assessed by Brown-Forsythe and Welch ANOVA, followed by Dunnett T3 test. A *p* value < 0.05 was considered as statistically significant.

## 3. Results

### 3.1. FMT from Aged Mice Causes Metabolic Changes in Young Mice

To examine the vascular and metabolic effects of age-associated dysbiosis, an aged-to-young FMT was conducted for 6 consecutive weeks (Figure 1A). After 1-week antibiotic depletion of background microbiome, the young recipient mice (Young (Control) and Young (FMT)) received FMT from either aged or age-matched young donor mice, which did not receive antibiotic pretreatment, with reference to documented protocols from previous studies [10,12]. C57BL/6 mice were chosen to understand the impact of aged microbiome on the young host and to minimize the influence of other background diseases. After the 6-week FMT, the body weights of young mice undergoing FMT (aged-transplanted; Young (FMT)) was lower than those of young-transplanted young mice (Young (Control)) (Figure 1B). During the 6-week FMT, in contrast to Young (Control) mice which showed increasing body weights, both Young (FMT) and aged mice (Aged) were losing body weights gradually (Figure 1C). Consistently, Aged and Young (FMT) mice exhibited a similar body weight percentage reduction during the 6-week FMT (Figure 1D). Besides, the amount of food intake was not significantly altered in Young (Control) and Young (FMT) during the 6 weeks (Figure S1A), hinting that body weight changes are most likely due to FMT. Additionally, the weights of certain organs (liver, spleen, kidney, and gastrocnemius) (Figure 1E), and adipose tissues, including inguinal subcutaneous adipose tissue (ingSAT), perigonadal visceral adipose tissue (pgVAT) and brown adipose tissue (BAT), of Young (FMT) mice were lower than those of Young (Control) mice (Figure 1F,G). After the 3rd week of FMT, ITT revealed insulin resistance in Aged and Young (FMT) mice (Figure S2). After the 6-week FMT, GTT (Figure 1H,I) and ITT (Figure 1J,K) showed glucose intolerance and insulin resistance in Aged and Young (FMT) mice. FMT slightly increased serum low-density lipoprotein (LDL) levels in Young (FMT) mice (Figure S1B–E).

### 3.2. FMT from Aged Mice Impairs Endothelial Function in Young Mice

**Figure 2:**
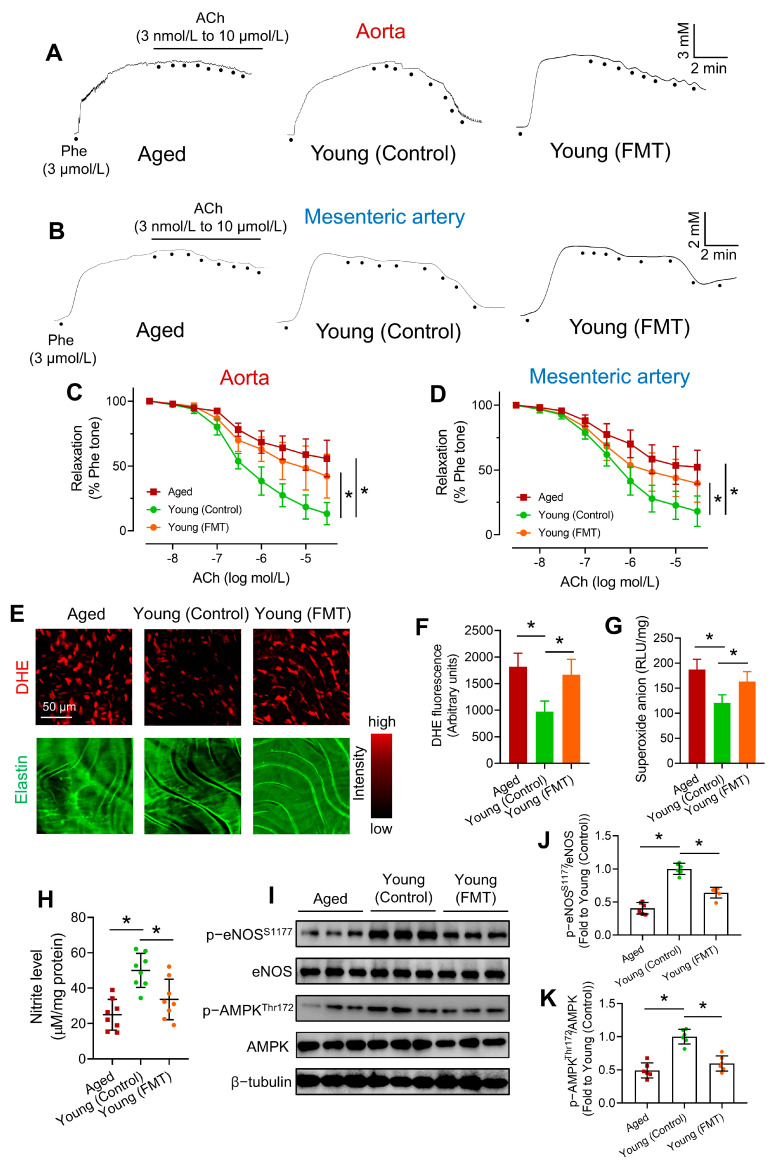
Effects of aged-to-young FMT on endothelial function. Representative traces for endothelium-dependent relaxations (EDRs) in (**A**) aortas and (**B**) mesenteric arteries of Aged, young-transplanted (Young (Control)) and aged-transplanted mice (Young (FMT)). Summary statistics of wire myography on EDRs in (**C**) aortas and (**D**) mesenteric arteries from different mouse groups. (**E**) Dihydroethidium (DHE) staining on en face endothelium of different mouse groups, and (**F**) corresponding quantification of DHE fluorescence. (**G**) Lucigenin-enhanced chemiluminescence on aortic ROS levels of different mouse groups. (**H**) Nitrite levels in aortas of different mouse groups. *N* = 8 per group. (**I**) Representative Western blots, and (**J**,**K**) quantification of Western blotting on expression of AMPK, p-AMPK at Thr172, eNOS and p-eNOS at Ser1177 in aortas of different mouse groups. *N* = 6 per group. Data are mean ± SD. * *p* < 0.05; Brown-Forsythe and Welch ANOVA and Dunnett T3 test.

To determine the effect of age-associated dysbiosis on the vasculature, we evaluated the endothelial function of arterial tissues by wire myography. Briefly, the arterial tissues were pre-contracted by phenylephrine (Phe), followed by cumulative additions of acetylcholine (ACh) to compare endothelium-dependent relaxations (EDRs) among different mouse groups [16]. ACh-induced EDRs in Phe-precontracted aortas of Aged and Young (FMT) mice were significantly attenuated when compared to those of Young (Control) mice (Figure 2A,C). By contrast, sodium nitroprusside (SNP)-induced endothelium-independent relaxations were comparable among 3 groups (Figure S3), indicating that the impaired vascular function was mainly due to endothelial dysfunction, but not endothelium-independent relaxations. Additionally, blunted ACh-indued relaxations in mesenteric arteries of Aged and Young (FMT) mice were observed (Figure 2B,D). Notably, the relaxations of mesenteric arteries are dependent on both endothelial nitric oxide synthase (eNOS)-derived NO and endothelium-derived hyperpolarizing factor (EDHF). N^G^-nitro-L-arginine methyl ester (L-NAME) was used to eliminate eNOS-derived NO production for determining the role of EDHF in ACh-induced relaxations of mesenteric arteries. Upon L-NAME incubation, EDHF-induced relaxations in mesenteric arteries were similar among the 3 mouse groups (Figure S4), indicating that impaired relaxations in mesenteric arteries were mainly due to reduced NO bioavailability. Nevertheless, our findings cannot completely rule out the possibility that aged microbiome might alter EDHF-induced relaxations in mesenteric arteries.

Endothelial dysfunction is often featured with ROS overproduction and diminished NO generation [22], we therefore measured ROS and NO levels in aortas. Both DHE staining (Figure 2E,F) and lucigenin-enhanced chemiluminescence assay (Figure 2G) showed elevated levels of ROS (mainly superoxide anions) in aortas of Aged and Young (FMT) mice. Nitrite assay revealed lower nitrite levels in aortas of Aged and Young (FMT) mice (Figure 2H), indicating decreased NO production. eNOS activation by phosphorylation at serine 1177 results in higher level of eNOS-derived NO [23]. Aged-to-young FMT suppressed p-eNOS S1177 but not total eNOS level in aortas of Young (FMT) mice as shown by Western blotting (Figure 2I,J). Upstream to eNOS, activated AMPK (phosphorylation at threonine 172) phosphorylates eNOS at serine 1177 to promote NO production [24]. Importantly, aged-to-young FMT significantly downregulated p-AMPK Thr172 level in Young (FMT) mice (Figure 2I,K), highlighting a suppressed AMPK/eNOS signaling. There are age-dependent elevations in diastolic, mean, and systolic blood pressure of C57BL/6 mice [25]. Moreover, higher mean arterial pressures were observed in both Aged and Young (FMT) mice (Figure S5).

### 3.3. FMT from Aged Mice Induces Vascular Inflammation and Telomere Dysfunction

**Figure 3:**
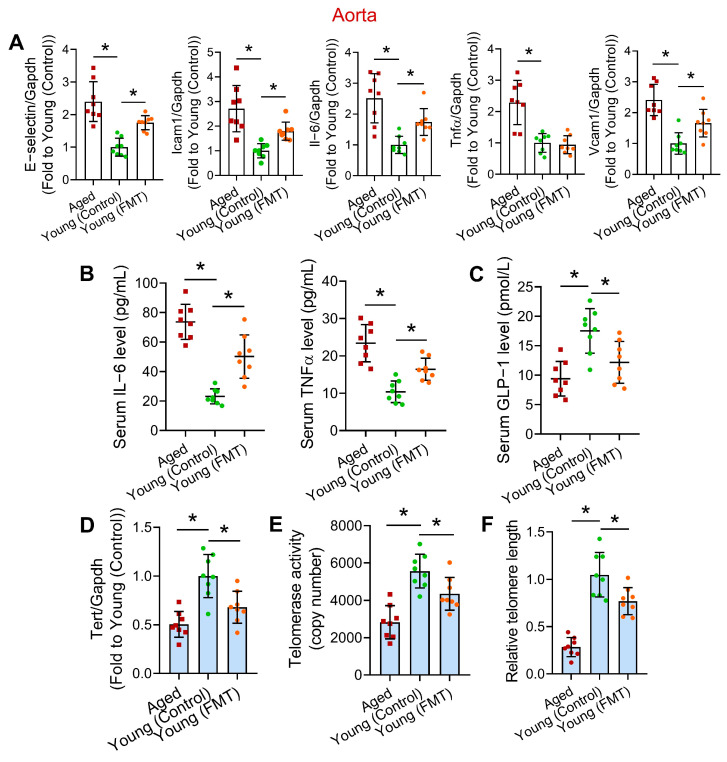
Effects of aged-to-young FMT on vascular and systemic inflammation, and vascular telomere function. (**A**) RT-PCR on mRNA levels of pro-inflammatory genes in aortas of Aged, young-transplanted (Young (Control)) and aged-transplanted mice (Young (FMT)). (**B**) ELISA on circulating inflammatory markers of different mouse groups. (**C**) ELISA on circulating GLP-1 levels of different mouse groups. (**D**) Tert mRNA level in aortas of different mouse groups. (**E**) Telomerase activities in aortas of different mouse groups. (**F**) Relative telomere length in aortas of different mouse groups. *N* = 8 per group. Data are mean ± SD. * *p* < 0.05; Brown-Forsythe and Welch ANOVA and Dunnett T3 test.

Upregulation of pro-inflammatory genes in the vasculature is a feature of vascular aging, and a vicious cycle exists between vascular oxidative stress and inflammation [26], so we wondered whether aged-to-young FMT promotes vascular inflammation. RT-PCR results showed that the expression of certain pro-inflammatory genes (i.e., E-selectin, Icam1, Il-6 and Vcam1) were significantly elevated in aortas of the Aged and Young (FMT) mice (Figure 3A), implying vascular inflammation. Since microbial alterations often cause shift in metabolite, peptide, and cytokine pools in host circulation to affect the vasculature [5], and aging is associated with chronic systemic inflammation and elevated levels of inflammatory cytokines in circulation [27], we conducted ELISA to measure the levels of certain circulating inflammatory markers upon aged-to-young FMT. Notably, circulating IL-6 and TNFα are two well-known inflammatory markers associated with higher vascular risk [28], and raised circulating levels of these cytokines are associated with age-related deterioration in body function [29]. Serum levels of IL-6 and TNFα were found higher in Aged and Young (FMT) mice (Figure 3B), hinting a pro-inflammatory vascular environment. As an intestine-derived peptide, GLP-1 confers vasoprotection by improving endothelial function and eliciting anti-oxidative and anti-inflammatory effects [30]. Notably, intestinal GLP-1 secretion declines with age [31]. We therefore suspected that aged microbiome might lower intestinal GLP-1 production. Notably, the serum level of GLP-1 was found lower in both Aged and Young (FMT) mice (Figure 3C).

Telomere dysfunction, particularly telomere shortening, is considered as a critical feature of cardiovascular aging, and putatively increases the risks of cardiovascular complications [32]. Assessing telomere function shall partially reveal the extent of vascular aging. Besides, we were curious about the potential interrelation between different aging hallmarks, such as dysbiosis and telomere attrition. Therefore, we sought to study whether aged-to-young FMT promotes vascular telomere dysfunction in young mice, by measuring level of telomerase reverse transcriptase (Tert), a catalytic subunit of telomerase, telomerase activity and relative telomere length in mouse aortas. RT-PCR results showed a downregulated Tert level in aortas of Aged and Young (FMT) mice when compared to Young (Control) mice (Figure 3D). Importantly, the telomerase activity in aortas of Aged and Young (FMT) mice were lower than those in Young (Control) mice (Figure 3E). Furthermore, the relative telomere length in aortas of Aged and Young (FMT) mice were shown significantly shorter (~20–30%) than that of Young (Control) mice, although that of Young (FMT) mice was still higher than that of Aged mice (Figure 3F). We also evaluated the telomere function in other organs (i.e., heart, lung, liver, and kidney) of 3 mouse groups. However, despite a slightly lower Tert expression in hearts of Young (FMT) mice than Young (Control) mice, 6-week FMT did not greatly alter Tert expression, telomerase activity and relative telomere length in these organs of Young (FMT) mice (Figure S6).

### 3.4. Age-Associated FMT Triggers Intestinal Telomere Dysfunction and Breakdown of Intestinal Barrier Integrity

**Figure 4:**
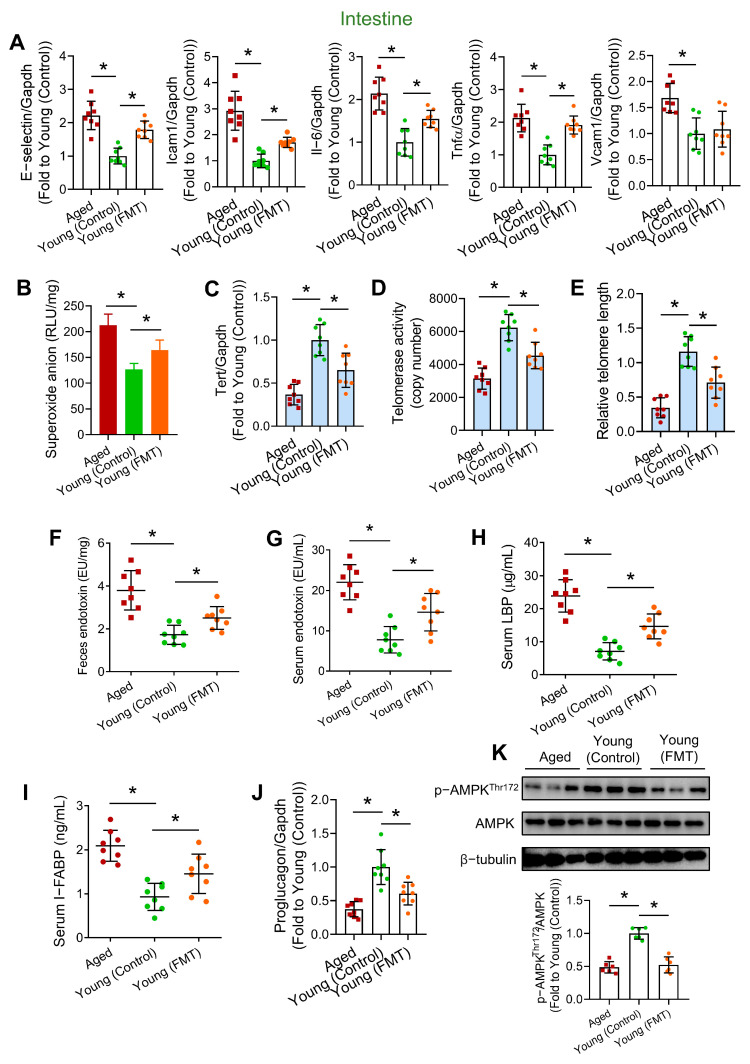
Effects of aged-to-young FMT on intestinal inflammation, telomere function and barrier function. (**A**) RT-PCR on mRNA levels of pro-inflammatory genes in intestines of Aged, young-transplanted (Young (Control)) and aged-transplanted mice (Young (FMT)). (**B**) Lucigenin-enhanced chemiluminescence on intestinal ROS levels of different mouse groups. (**C**) Tert mRNA level in intestines of different mouse groups. (**D**) Telomerase activities in intestines of different mouse groups. (**E**) Relative telomere length in intestines of different mouse groups. Endotoxin levels in (**F**) feces and (**G**) sera of different mouse groups. ELISA on serum levels of (**H**) LBP and (**I**) I-FABP of different mouse groups. (**J**) Proglucagon mRNA level in intestines of different mouse groups. *N* = 8 per group. (**K**) Representative Western blots and quantification of Western blotting on expression of AMPK and p-AMPK at Thr172 in intestines of different mouse groups. *N* = 6 per group. Data are mean ± SD. * *p* < 0.05; Brown-Forsythe and Welch ANOVA and Dunnett T3 test.

Since the intestine of the digestive system is one of the organs that firstly experiences and harbors the dysfunctional microbiome from aged donor mice, we suspected that the large intestines of the young recipient mice might undergo inflammation, oxidative damage, and premature telomere dysfunction. The intestinal expression of pro-inflammatory markers (i.e., E-selectin, Icam1, Il-6 and TNFα), along with intestinal oxidative stress, were found higher in Aged and Young (FMT) mice (Figure 4A,B). Premature telomere dysfunction was noted in intestinal tissues of Young (FMT) mice, as reflected by lower Tert expression (Figure 4C), decreased telomerase activity (Figure 4D) and shorter relative telomere length (~30–50% shorter) (Figure 4E). It is reasonable to postulate that certain harmful factors released by the dysfunctional microbiome cause the undesirable consequences in intestines. Compared to the Young (Control) mice, the fecal endotoxin levels in Aged and Young (FMT) mice were found remarkably higher (Figure 4F). We subsequently doubted the presence of endotoxemia upon aged-to-young FMT. Importantly, serum levels of endotoxin and LBP in Aged and Young (FMT) mice were found significantly higher than those in Young (Control) mice (Figure 4G,H), hinting impaired intestinal integrity. Specifically expressed by epithelial cells of the mucosal layer in intestine, I-FABP is considered as a biomarker for intestinal barrier dysfunction [21]. Later identification of high amounts of I-FABP in sera of Aged and Young (FMT) mice indicated increased intestinal permeability upon aged-to-young FMT (Figure 4I). Besides, the lower expression of intestinal proglucagon, a precursor to GLP-1, in Aged and Young (FMT) mice partially accounted for the diminished levels of circulating GLP-1 (Figure 4J).

### 3.5. Age-Associated FMT Leads to Microbial Shifts in Young Mice

To investigate age-related changes in the gut microbiome and the impact of FMT, we conducted 16S rRNA sequencing of Aged, Young (Control) and Young (FMT) mice in our study. Since FMT might not fully ensure engraftment of all microbial species from donor mice (e.g., aged and young donor mice) in recipient mice, the gut microbiome analysis data serves two major purposes. Firstly, they serve as a verification that the recipient mice have successfully harbored the aged microbiome. Secondly, the data somehow show the proportion of microbiome that were successfully engrafted in the recipient mice, where the engrafted proportion could already cause the phenotypic and genotypic changes in metabolic and aging hallmarks of recipient mice, providing insights to future studies on the role of successfully engrafted microbial species on aging process.

**Figure 5:**
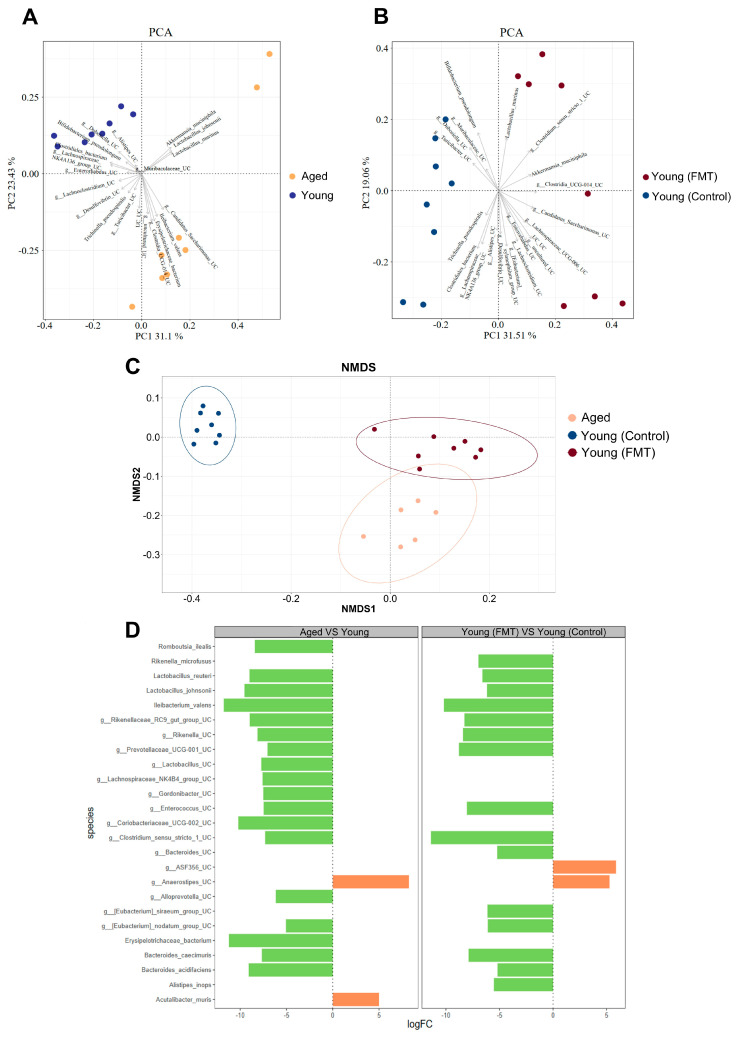
Effects of aged-to-young FMT on gut microbial profiles in young host mice. (**A**) Principal component analysis (PCA) plot revealing distinct clusters for fecal microbiome samples obtained from young (depicted in blue) and aged (in brown) mice before antibiotic treatment and FMT, highlighting the species contributing to this clustering. *N* = 8 per group. (**B**) PCA plot showing the clustering of fecal microbiome samples from young-transplanted (Young (Control); depicted in blue) and aged-transplanted mice (Young (FMT); in red). *N* = 8 per group. (**C**) Non-Metric Multi-Dimensional Scaling (NMDS) plot displaying the clustering of microbiome across various mouse groups. *N* = 6–8 per group. (**D**) Differential abundance analysis on the mean difference in centered log ratio for enriched species in young, aged, Young (Control) and Young (FMT) mice. *N* = 8 per group.

Before antibiotic treatment and FMT, we first established the baseline taxonomy of the fecal microbiome in both young and aged mice. As expected, we observed shifts in microbial beta diversity associated with age, a phenomenon previously documented in both mice [33], and humans [34]. Our analysis revealed specific species that contributed to the clustering of aged mice, such as *Ileibacterium*, *Erysipelotrichaceae bacterium*, *Candidatus Saccharimonas_UC*, *Akkermansia muciniphila*, *Clostridia_UCG-014_UC*, and *Lactobacillus murinus* (Figure 5A). A total of 18 species, such as *Bacteroides caecimuris*, *Enterococcus_Unclassified*, *Rikenellaceae_RC9_gut_group_Unclassified*, *Rikenella_Unclassified*, *Prevotellaceae_UCG-001_Unclassified*, *Ileibacterium valens*, *Clostridium_sensu_stricto_1_Unclassified*, *Erysipelotrichaceae bacterium* and others, exhibited significant enrichment in the aged versus young mice (Figure 5D).

Following FMT, we observed significant alterations in the gut microbial profiles of Young (FMT) mice, with the most substantial changes occurring at the “Post-FMT” time point. Interestingly, the gut microbial profile of Young (FMT) mice appeared to resemble that of Aged mice more closely when compared to Young (Control) mice (Figure 5C). This suggested that the intestinal environment of recipient mice played a crucial role in shaping the long-term success of microbiome engraftment. The clustering of Young (FMT) mice was driven by species, such as *Candidatus Saccharimonas_UC, Clostridia_UCG-014_UC, Enterorhabdus_UC, Akkermansia muciniphila, Lactobacillus murinus,* and *xylanophilum* (Figure 5B). Transplanting young mice with an aged microbiome led to the enrichment of signature species, many of which overlapped with those enriched in the gut microbiome of aged mice (Figure 5D).

The lower abundance of *Bifidobacterium pseudolongum* and *Dubosiella* might partially account for the enhanced intestinal inflammation in Young (FMT) mice (Figure 5B), where *Bifidobacterium pseudolongum* was previously shown to exert anti-inflammatory effects in intestine through increasing the production of secondary bile acids [35], and *Dubosiella* was previously found to be negatively correlated with intestinal mRNA levels of inflammatory markers (e.g., IL-1β, IL-6, and TNF-α) [36].

### 3.6. Age-Associated FMT Impairs Intestinal Function Through AMPK Inhibition

**Figure 6:**
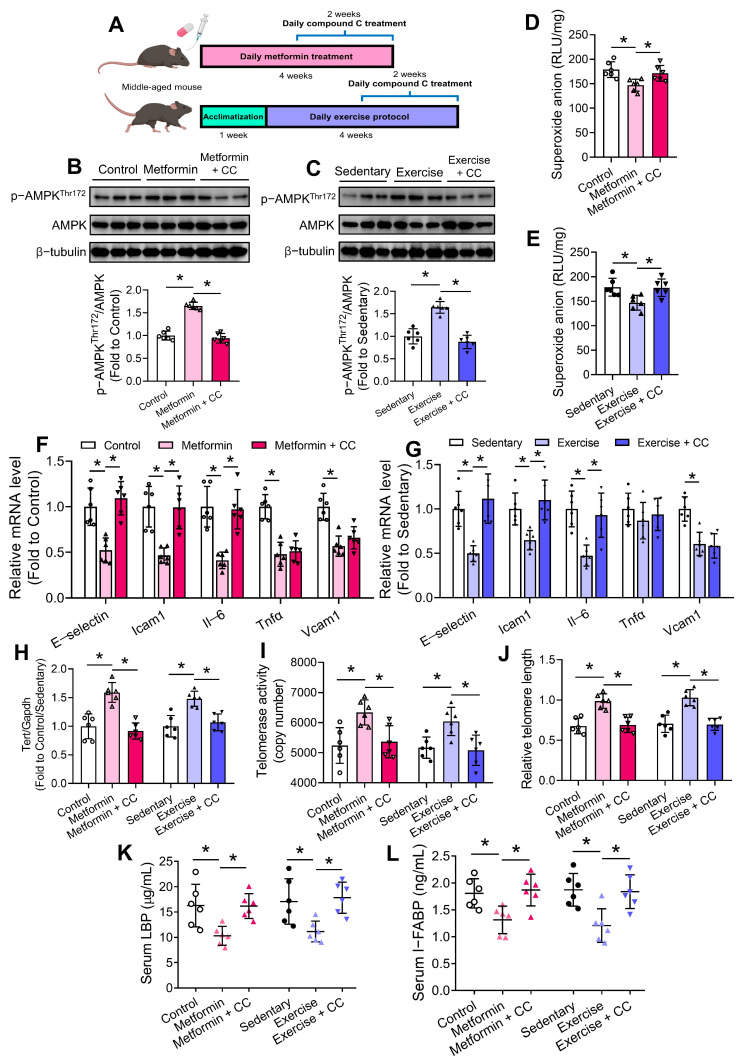
Effects of chronic metformin treatment and moderate exercise training on intestinal homeostasis. (**A**) Schematic diagram on chronic metformin treatment and moderate exercise training with the presence and absence of compound C (CC) treatment in middle-aged C57BL/6 mice. Representative Western blots and quantification of Western blotting on expression of AMPK and p-AMPK at Thr172 in intestines of (**B**) metformin-treated mice, and (**C**) exercise-trained mice. Lucigenin-enhanced chemiluminescence on intestinal ROS levels of (**D**) metformin-treated mice, and (**E**) exercise-trained mice. RT-PCR on mRNA levels of pro-inflammatory genes in intestines of (**F**) metformin-treated mice, and (**G**) exercise-trained mice. (**H**) Tert mRNA level in intestines of different mouse groups. (**I**) Telomerase activities in intestines of different mouse groups. (**J**) Relative telomere length in intestines of different mouse groups. ELISA on serum levels of (**K**) LBP and (**L**) I-FABP of different mouse groups. *N* = 6 per group. Data are mean ± SD. * *p* < 0.05; Brown-Forsythe and Welch ANOVA and Dunnett T3 test.

Deregulated nutrient-sensing is another aging hallmark [2], and AMPK pathway is one of the critical energy-sensing pathways [37], where its inactivation has been noted in mouse aortas upon age-to-young FMT (Figure 2I). We were interested in the potential interrelation between different aging hallmarks, like dysbiosis and deregulated nutrient-sensing. Besides, intestinal barrier dysfunction was identified upon aged-to-young FMT (Figure 4F–I), and AMPK is a critical regulator of intestinal permeability [38]. We therefore also measured AMPK activation in mouse intestinal tissues. Western blotting results showed that intestinal p-AMPK levels were suppressed in both Aged and Young (FMT) mice (Figure 4K). To investigate the role of AMPK in age-associated intestinal dysfunction, some middle-aged mice (38–40 weeks old) were subjected to chronic metformin treatment and exercise training (Figure 6A), where both interventions are well-known for activating AMPK [39]. Middle-aged mice, rather than aged mice, were chosen because of better performance on exercise protocol and less vulnerability to experimental procedures, in the accordance of the 3Rs principle (Replacement, Reduction and Refinement). FMT-receiving young mice were not chosen for metformin treatment and exercise training because of the presence of too many confounding variables (antibiotics, FMT, metformin/exercise, and compound C) for drawing straightforward conclusions, and the purpose of minimizing pain and suffer of animals. To determine whether these two interventions also activated AMPK in intestine, we measured the protein levels of AMPK and p-AMPK. Western blotting results showed upregulated p-AMPK levels in mouse intestines upon metformin treatment and exercise training, where co-treatment of the AMPK inhibitor compound C ablated such effects (Figure 6B,C). Moreover, both interventions caused the suppression of oxidative stress as shown by lucigenin-enhanced chemiluminescence assay (Figure 6D,E), and downregulation of certain pro-inflammatory genes in mouse intestines, whereas compound C co-treatment reversed such effects (Figure 6F,G).

Notably, metformin treatment but not exercise training increased intestinal Tert expression, while compound C co-treatment significantly suppressed Tert levels in both cases (Figure 6H). Importantly, both interventions increased the telomerase activity and relative telomere length, where compound C ablated such beneficial effects (Figure 6I,J). Both interventions lowered serum levels of LBP and I-FABP through AMPK activation (Figure 6K,L), implying inhibited endotoxemia and improved intestinal barrier function. Furthermore, both interventions upregulated intestinal proglucagon expression and circulating GLP-1 level, potentially also via AMPK activation (Figure S7).

## 4. Discussion

In humans and rodents, aging is associated with a gradual decline in body weights especially in old age [40]. Surprisingly, aged-to-young FMT caused a decline in body weights without altering the amount of food intake, hinting that age-associated dysbiosis mainly accounted for the body weight change. The decreased weights of organs and adipose tissues contributed to the overall weight loss, implying a systemic effect of age-associated FMT on metabolism of young recipient mice. The significantly and gradually altered blood glucose profiles, and slightly changed serum lipid profiles partially reflect the premature metabolic impairment in Young (FMT) mice.

The alterations in blood molecular profiles (e.g., glucose and lipids) prompted a reasonable postulation that the homeostasis of endothelium, the innermost layer of the vasculature exposing to various circulating factors, might be affected. Hyperglycemia and dyslipidemia promote the development of endothelial dysfunction. Wire myograph results suggested endothelial dysfunction in aortas and mesenteric arteries of Young (FMT) mice. High oxidative stress, lower NO bioavailability and dysregulated AMPK/eNOS axis all contribute to endothelial dysfunction [41]. Since hydrogen sulfide (H_2_S) and NO are mutually dependent during the regulation of EDRs [42], we cannot exclude the possibility that age-associated dysbiosis lowers NO bioavailability and triggers vascular dysfunction through a H_2_S-dependent manner. Future efforts are required to understand the comprehensive role of H_2_S in age-associated dysbiosis and vascular aging. Upregulation of pro-inflammatory genes in aortic tissues implied vascular inflammation, which also exacerbates vascular oxidative stress and endothelial dysfunction [43]. It is therefore rational to postulate that endothelial cells might be exposed to increased levels of harmful factors, particularly those related to inflammatory regulation, and decreased levels of beneficial factors upon age-associated FMT.

Notably, ELISA results suggested elevated levels of circulating inflammatory markers, representing potential accelerators on endothelial dysfunction, and implying systemic inflammation upon FMT. As a gut-derived peptide, GLP-1 elicits glucoregulatory effects to reduce excessive levels of blood glucose, and vasoprotective effects to improve endothelial function through AMPK activation and ROS counteraction [44]. Lower circulating GLP-1 levels partially contributed to endothelial dysfunction, elevated vascular oxidative stress, and increased arterial pressure [45] in Young (FMT) mice. However, the magnitude of decline in GLP-1 levels might be insufficient to fully address the significantly altered vascular function and blood pressure that the participation of other factors should not be overlooked. Further molecular profiling shall facilitate the identification of more key molecules involved in the gut-vascular connection.

Upon aged-to-young FMT, telomere dysfunction was observed in both aortic and intestinal tissues of Young (FMT) mice, partially indicative to premature vascular and intestinal aging, since telomere dysfunction is often considered the primary cause of aging [46]. The extent of telomere shortening was slightly higher in intestines (~30–50%) than in aortas (~20–30%). Whether the proliferative capacity of vascular cells (e.g., endothelial cells) greatly alters upon dysbiosis-resulted vascular injury requires further study. Although telomere dysfunction was not observed in other organs after the 6-week FMT, we cannot exclude the possibility that such dysfunction might be triggered in other organs upon prolonged exposure to dysbiotic microbiome. In other words, gut-vascular dysfunction might represent an early event preceding systemic impact induced by aged microbiome. Besides, we cannot exclude the possibility that the sensitivity to harmful factors upon age-associated dysbiosis and the mechanism of telomere regulation might be different in distinct organs/tissues/cells. Whether intestine is more resistant to dysbiosis-induced telomere dysfunction, even in the closest proximity to fecal microbiome, requires further investigation. Notably, our results on telomere dysfunction in bulk mouse vascular tissues correlate with a previous report that telomere dysfunction in endothelial cells amplifies inflammatory signaling and oxidative stress to impair endothelial function [47]. Future studies can investigate the contributory roles and detailed mechanism of telomere dysfunction in different vascular cells, like endothelial cells and vascular smooth muscle cells, separately during age-associated dysbiosis and vascular aging.

Additional to telomere dysfunction, aged-to-young FMT caused intestinal oxidative stress and inflammation, and elevated fecal endotoxin level, potentially attributed to the shifted microbial profile towards that of aged mice. Age-to-young FMT also induced intestinal barrier dysfunction and endotoxemia, which are believed to promote systemic and cardiovascular inflammation [48]. Meanwhile, intestinal inflammation and increased intestinal permeability are often concurrent events aggravating endotoxemia [49], which further drives endothelial dysfunction and vascular inflammation [50]. Importantly, endotoxemia was found positively correlated to metabolic impairment, particularly disrupted glucose homeostasis and hyperlipidemia [51]. The aged microbiome promoted endotoxemia by increasing intestinal permeability and reduced intestinal production of GLP-1 to exacerbate vascular dysfunction.

We next sought to understand the mechanism by how age-related microbial alterations triggered telomere dysfunction and phenotypic changes in intestines. Our results showed that age-associated dysbiosis suppressed, while chronic metformin treatment and moderate exercise promoted intestinal AMPK activation. Through AMPK activation, both interventions partially reversed intestinal oxidative stress and inflammation, telomere dysfunction, intestinal barrier dysfunction and GLP-1 downregulation in middle-aged mice. AMPK activation was previously shown to improve intestinal barrier function [38], and intestinal metformin-AMPK axis contributes to the regulation of glucose homeostasis and body weight [52]. AMPK inactivation in intestines might partially account for metabolic impairment in Aged and Young (FMT) mice. Responsiveness of AMPK signaling generally declines with aging [53], and declined AMPK activity was found in aged vasculature [26]. Our findings provided evidence that AMPK activity decreased in aged intestine, and transfer of dysbiotic microbiome from aged mice suppressed intestinal AMPK activity, potentially triggering subsequent harmful consequences in intestine and vasculature.

Previously, AMPK activation by metformin was shown to inhibit intestinal inflammation [54]. We provided clues that moderate exercise also elicited anti-inflammatory effect on intestine through AMPK activation. However, AMPK inhibition did not fully ablate the anti-inflammatory effects of metformin and exercise, hinting that both interventions suppress intestinal inflammation through multiple mechanisms. Additionally, we found that AMPK activation was upstream to ROS alleviation and telomere regulation in intestine upon metformin treatment and exercise training. Our findings extended the beneficial mechanisms of metformin and exercise in counteracting intestinal and vascular abnormalities during aging. Previously, AMPK activation was shown to be upstream to TERT transcription in tumor cells [55]. As a critical cellular energy sensor, AMPK is involved in various signaling pathways that the detailed mechanism on how AMPK regulates telomere function in different systems require future extensive study. We cannot rule out the possibility that AMPK regulates telomere function through SIRT1 signaling, tightly related to telomere maintenance [56], and through its anti-inflammatory effect since telomeres are sensitive to inflammation [57]. High levels of gut-derived endotoxins and chronic inflammation were clinically linked to telomere shortening [58], potentially accounting for vascular telomere dysfunction upon age-associated dysbiosis.

Extensive efforts are still needed to identify the groups of commensal and pathogenic bacteria that contribute to the regulation of host telomere function in different cells. Most importantly, our findings broaden our understanding towards the sophisticated network among multiple hallmarks of aging, including dysbiosis, deregulated nutrient-sensing (e.g., AMPK inactivation), chronic inflammation and telomere attrition, in terms of gut-vascular connection. Further efforts are required aiming to explore the interrelation among aging hallmarks in different age-associated diseases and identify interventions that potentially influence the interrelation.

## 5. Conclusions

**Figure 7:**
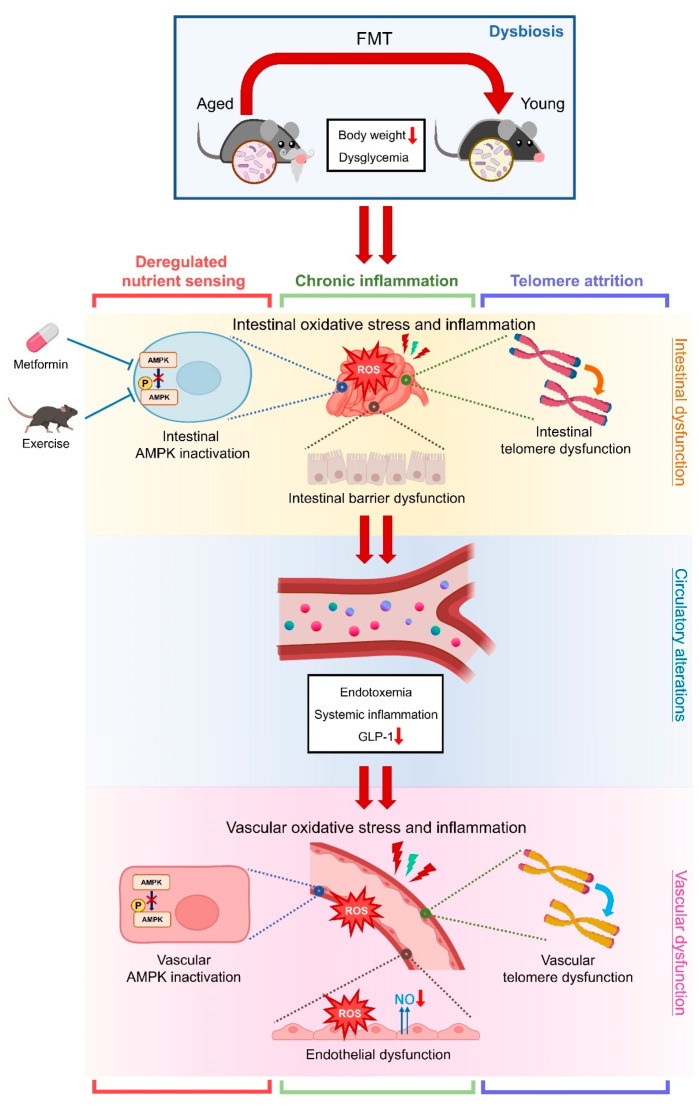
Schematic overview of the study. Aged microbiome induces metabolic impairments and vascular dysfunction in young mice. Aged microbiome causes telomere dysfunction, oxidative stress, and inflammation in intestines and vasculature of young mice. Metformin and moderate exercise potentially retard hallmarks of intestinal aging through AMPK activation. The study highlights the network among multiple aging hallmarks, including dysbiosis, deregulated nutrient sensing, chronic inflammation and telomere attrition, in terms of gut-vascular connection.

In summary, we demonstrate that age-associated dysbiosis, achieved by aged-to-young FMT, caused endothelial dysfunction, telomere dysfunction, inflammation, and oxidative stress in vascular tissues, partially reflective of premature vascular aging. Moreover, aged-to-young FMT also caused metabolic impairment and altered gut microbial profiles in Young (FMT) mice, along with disrupted intestinal integrity. Interestingly, aged-to-young FMT caused telomere dysfunction in both intestinal and aortic tissues. Metformin and moderate exercise might potentially retard intestinal aging through AMPK activation. These findings highlight the harmful effects of aged microbiome on intestine and vasculature, providing important insight that gut-vascular connection represents a potential intervention target against age-related cardiometabolic complications (Figure 7). Altogether, this study deepens our understanding towards the comprehensive network underlying different hallmarks of aging and provides preventive insights on vascular and intestinal aging.

## Data Availability

The data that support the findings of this study are either included in the Manuscript and Supplementary Materials or available from the corresponding author upon reasonable request.

## Supplementary Materials

The following supporting information can be downloaded at: https://www.mdpi.com/article/10.3390/antiox13101250/s1, Figure S1. Effects of aged-to-young FMT on food intake and lipid profiles; Figure S2. Effects of 3-week aged-to-young FMT on glucose homeostasis; Figure S3. Effects of aged-to-young FMT on endothelium-independent vasodilation in aortas; Figure S4. Effects of age-associated FMT on EDHF-dependent vasodilation in mesenteric arteries; Figure S5. Effect of age-associated FMT on arterial pressure; Figure S6. Effects of age-associated FMT on telomere function of indicated organs; Figure S7. Effects of metformin treatment and exercise training on GLP-1 level; Table S1. Primer list for quantitative RT-PCR.
