# Supplementary material for: Aged Gut Microbiome Induces Metabolic Impairment and Hallmarks of Vascular and Intestinal Aging in Young Mice
Source: Antioxidants (Basel). 2024 Oct 17;13(10):1250. doi: 10.3390/antiox13101250 (PMC11505429; doi:10.3390/antiox13101250)
Supplement: Supplementary file 1 [file antioxidants-13-01250-s001.zip › antioxidants-3244123-supplementary.pdf]

# Supplementary Materials

## Aged gut microbiome induces metabolic impairment and hallmarks of vascular and intestinal aging in young mice

Chak Kwong Cheng[1,#,*], Lianwei Ye[2,#], Yuanyuan Zuo[3], Yaling Wang[4], Li Wang[1], Fuyong Li[2], Sheng Chen[4], Yu Huang[1,*]

[1]Department of Biomedical Sciences, City University of Hong Kong, Hong Kong SAR, China
[2]Department of Infectious Diseases and Public Health, Jockey Club College of Veterinary Medicine and Life Sciences, City University of Hong Kong, Hong Kong SAR, China
[3]School of Biomedical Sciences, The Chinese University of Hong Kong, Hong Kong SAR, China
[4]Department of Food Science and Nutrition, The Hong Kong Polytechnic University, Hong Kong SAR, China

[#]Chak Kwong Cheng and Lianwei Ye contributed equally to this study.
***Correspondence should be addressed to:** Yu Huang and Chak Kwong Cheng, City University of Hong Kong, Tat Chee Avenue, Hong Kong SAR 999077, China. Email: yu.huang@cityu.edu.hk and superandy333@gmail.com.

**Part I: Supplementary figures**

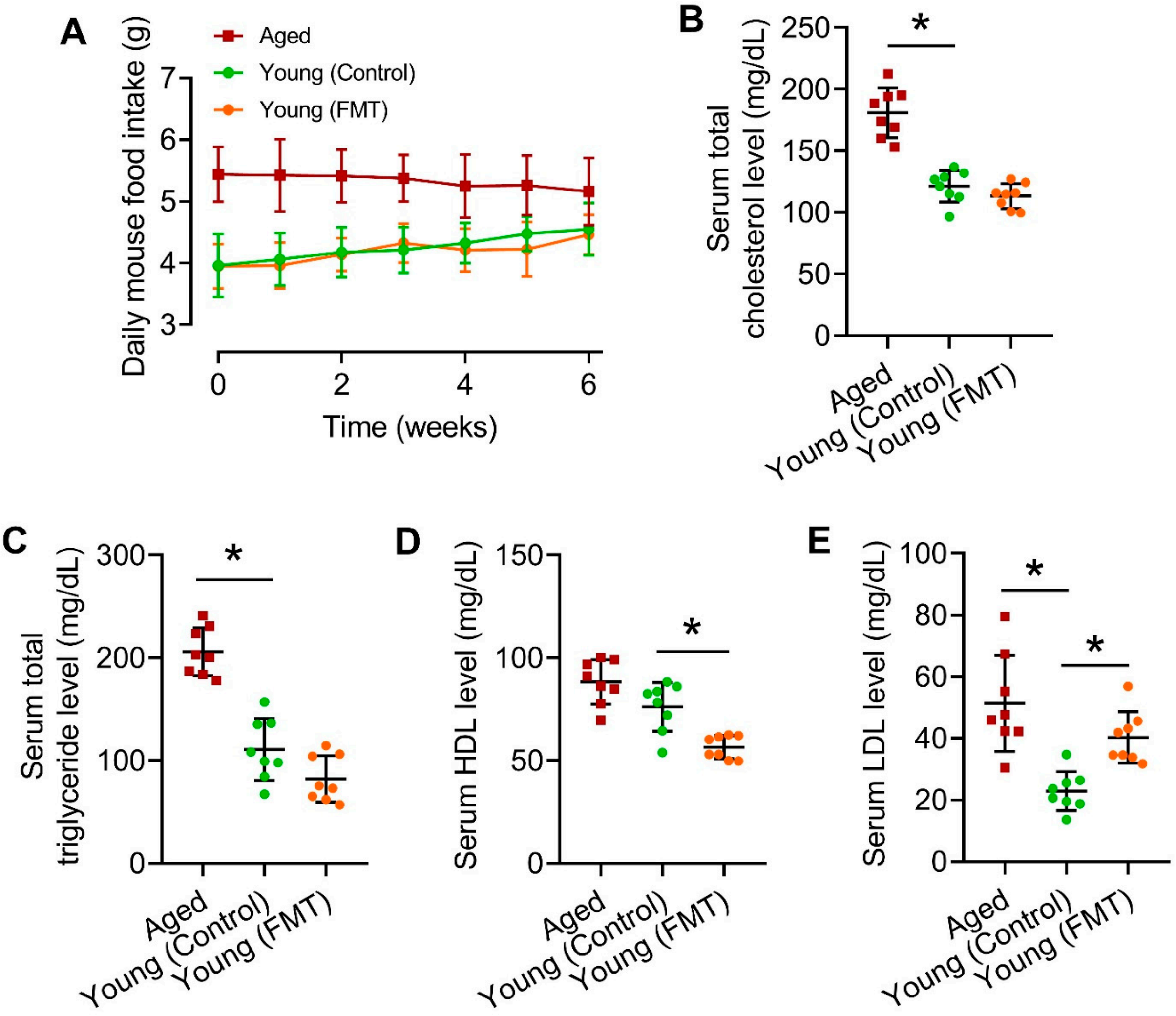


**Figure S1.** Effects of aged-to-young FMT on food intake and lipid profiles. **(A)** Changes in daily food intake of Aged, young-transplanted (Young (Control)) and aged-transplanted mice (Young (FMT)) during the 6-week FMT. Serum levels of **(B)** total cholesterol, **(C)** total triglycerides, **(D)** high-density lipoprotein (HDL) cholesterol, and **(E)** low-density lipoprotein (LDL) cholesterol in different mouse groups. $N = 8$ per group. Data are mean ± SD. *p<0.05; Brown-Forsythe and Welch ANOVA and Dunnett T3 test.

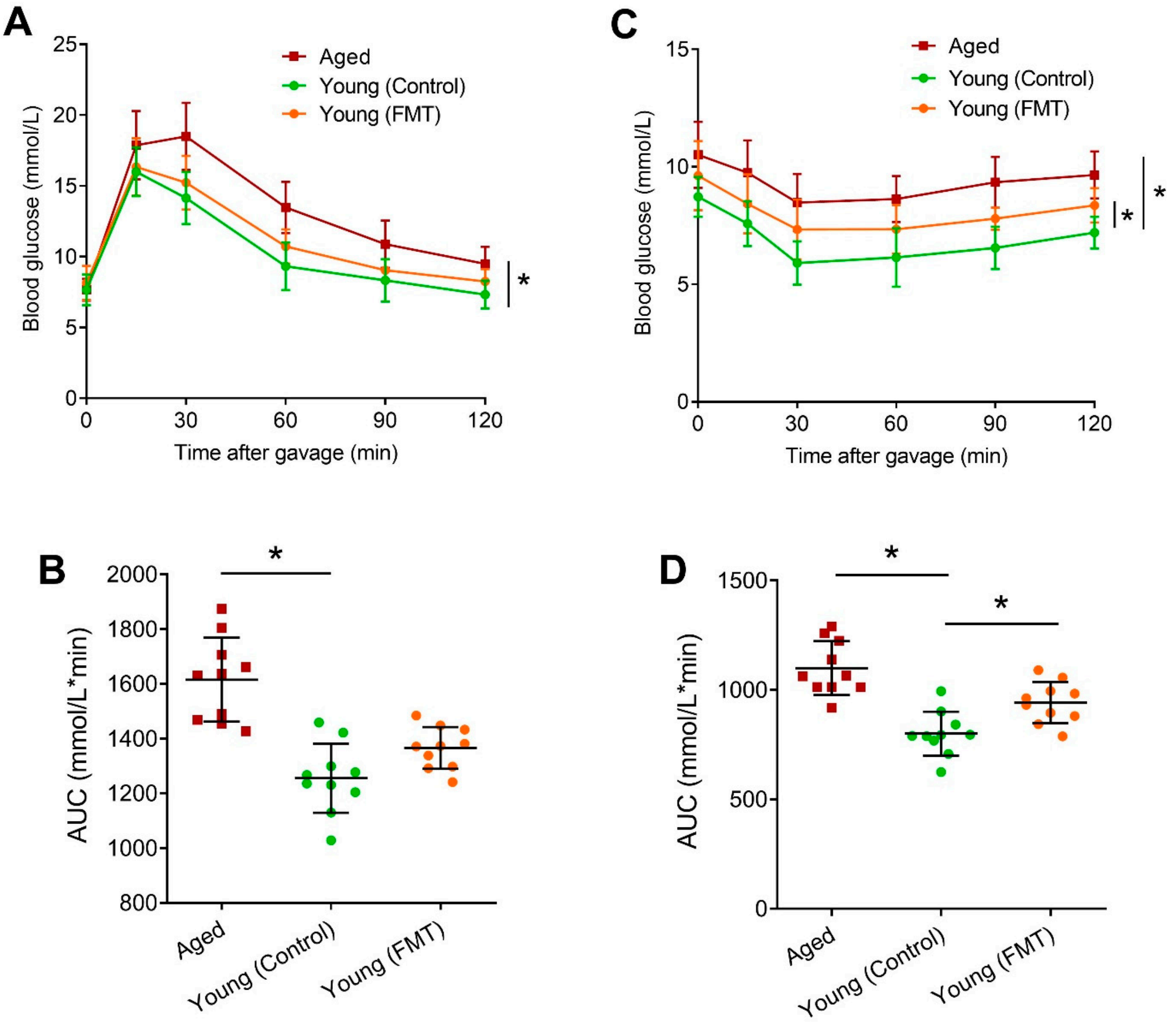


**Figure S2.** Effects of 3-week aged-to-young FMT on glucose homeostasis. **(A)** Glucose tolerance test (GTT) on mice at week 3 of FMT, and **(B)** corresponding area under curve (AUC) analysis of glucose over time. **(C)** Insulin tolerance test (ITT) of mice at week 3 of FMT, and **(D)** corresponding AUC analysis of glucose over time. *N* = 10 per group. Data are mean ± SD. *p<0.05; Brown-Forsythe and Welch ANOVA and Dunnett T3 test.

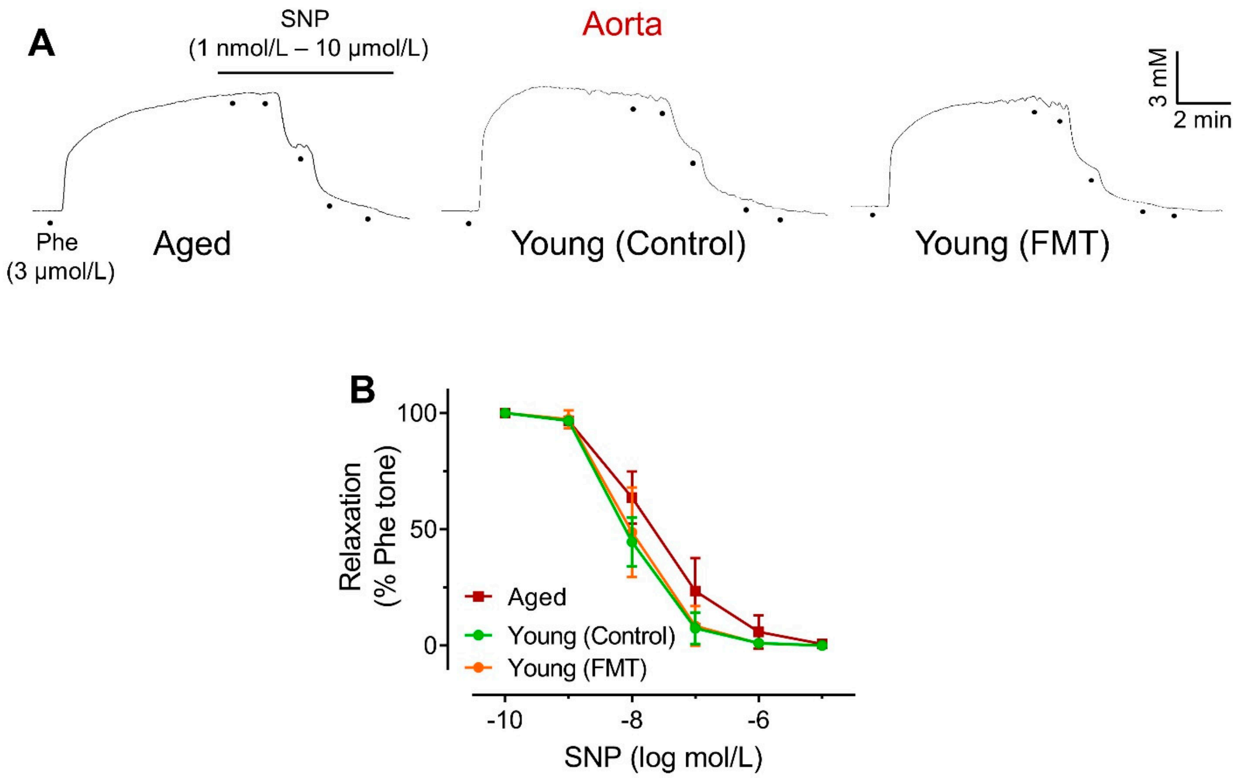


**Figure S3.** Effects of aged-to-young FMT on endothelium-independent vasodilation in aortas. **(A)** Representative traces for sodium nitroprusside (SNP)-induced endothelium-independent relaxations in aortas of Aged, young-transplanted (Young (Control)) and aged-transplanted mice (Young (FMT)). **(B)** Summary statistics of wire myography on endothelium-independent relaxations in aortas of different mouse groups. *N* = 8 per group. Data are mean ± SD. Brown-Forsythe and Welch ANOVA and Dunnett T3 test.

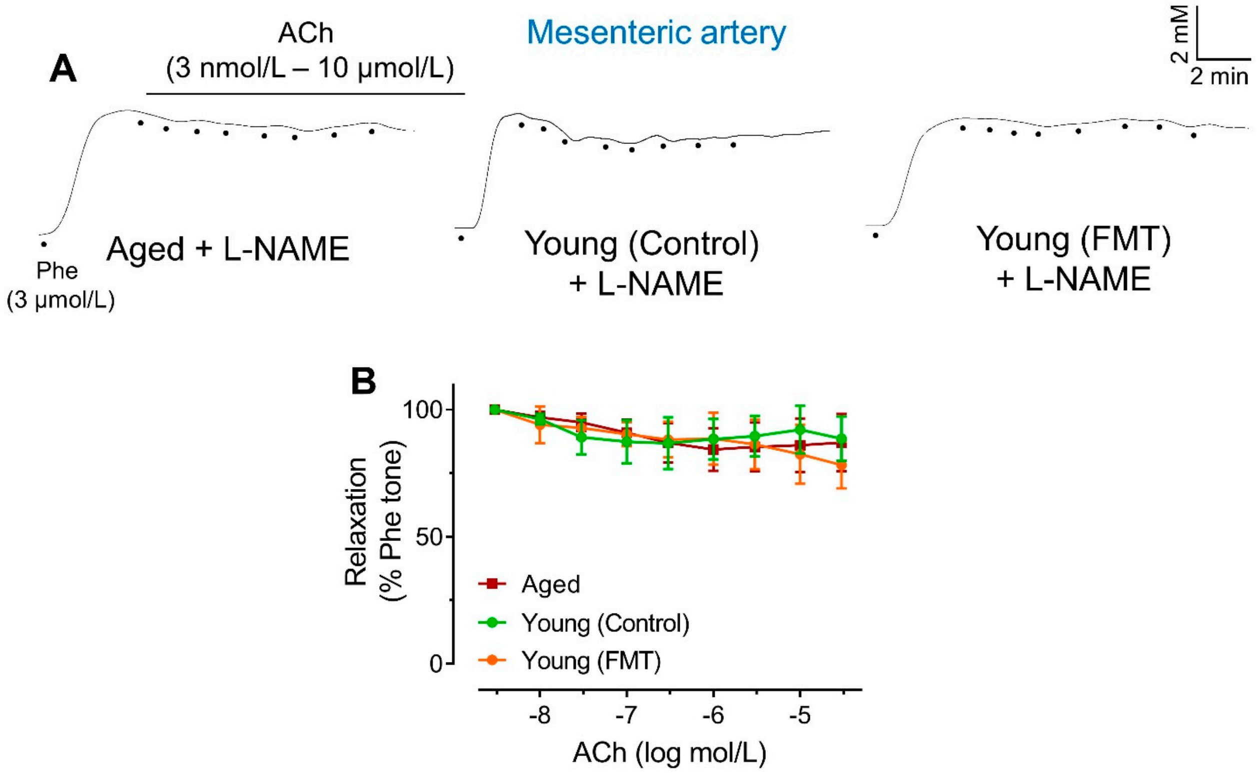


**Figure S4.** Effects of age-associated FMT on EDHF-dependent vasodilation in mesenteric arteries. **(A)** Representative traces for endothelium-dependent relaxations (EDRs) in mesenteric arteries of Aged, young-transplanted (Young (Control)) and aged-transplanted mice (Young (FMT)) upon NG-nitro-L-arginine methyl ester (L-NAME) incubation. **(B)** Summary statistics of wire myography on EDRs in mesenteric arteries of different mouse groups upon L-NAME incubation. *N* = 8 per group. Data are mean ± SD. Brown-Forsythe and Welch ANOVA and Dunnett T3 test.

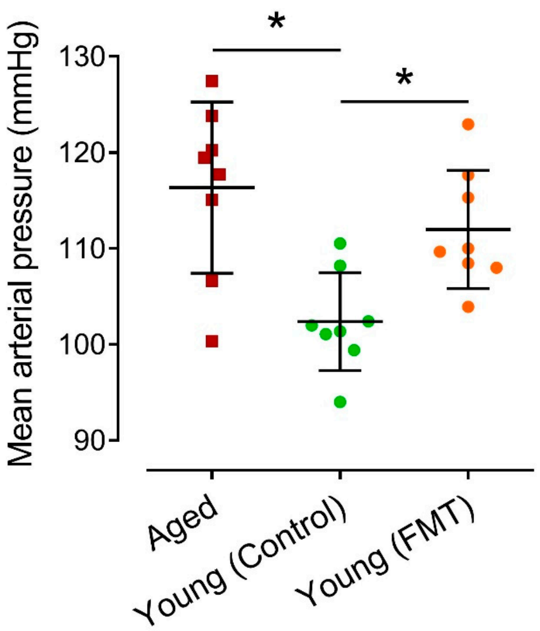


**Figure S5.** Effect of age-associated FMT on arterial pressure. Mean arterial pressure of Aged, young-transplanted (Young (Control)) and aged-transplanted mice (Young (FMT)) after the 6-week FMT. *N* = 8 per group. Data are mean ± SD. *p<0.05; Brown-Forsythe and Welch ANOVA and Dunnett T3 test.

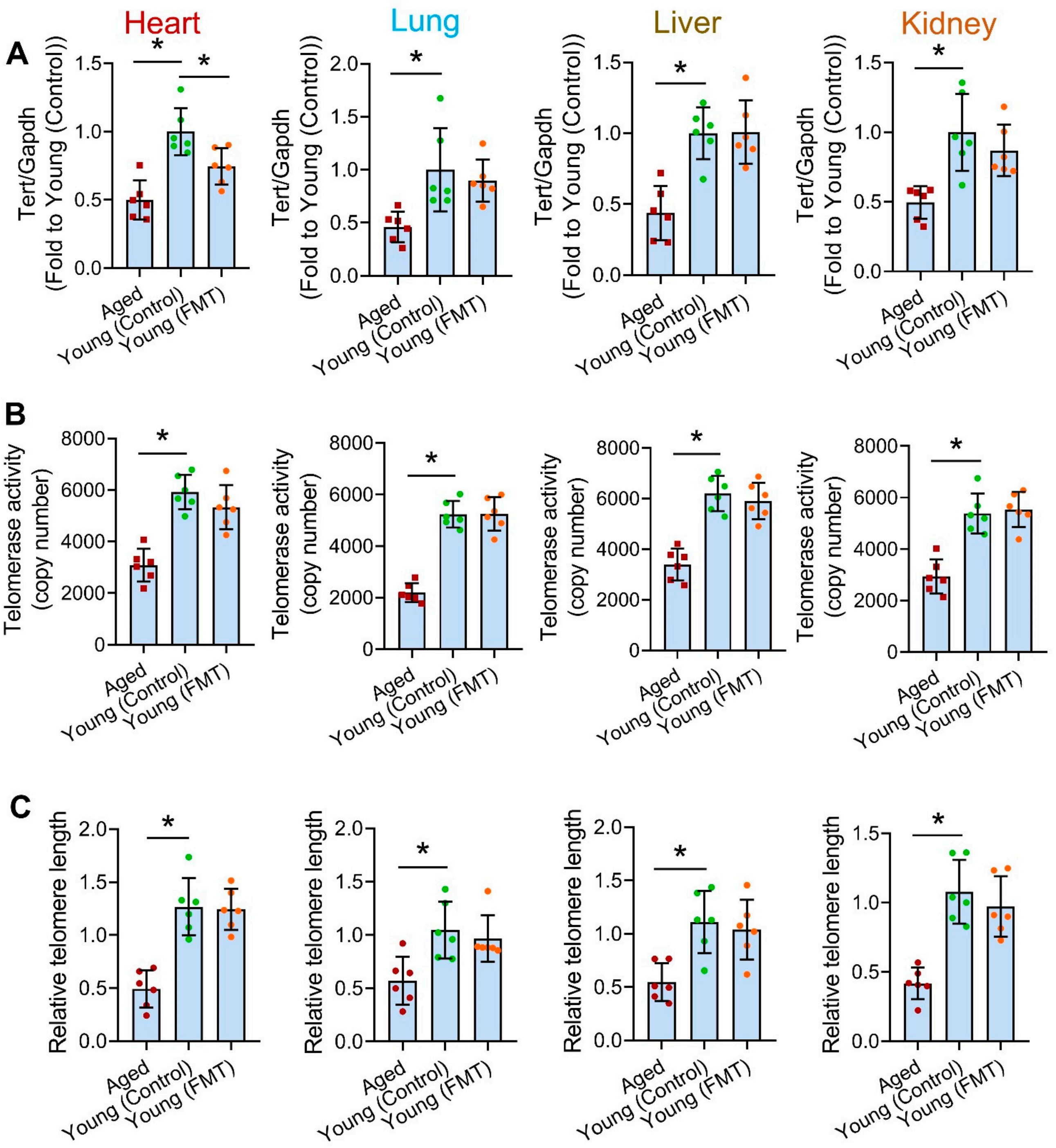


**Figure S6.** Effects of age-associated FMT on telomere function of indicated organs. **(A)** Tert mRNA level, **(B)** telomerase activities, and **(C)** relative telomere length in hearts, lungs, livers, and kidneys of Aged, young-transplanted (Young (Control)) and aged-transplanted mice (Young (FMT)). *N* = 6 per group. Data are mean ± SD. *$p<0.05$; Brown-Forsythe and Welch ANOVA and Dunnett T3 test.

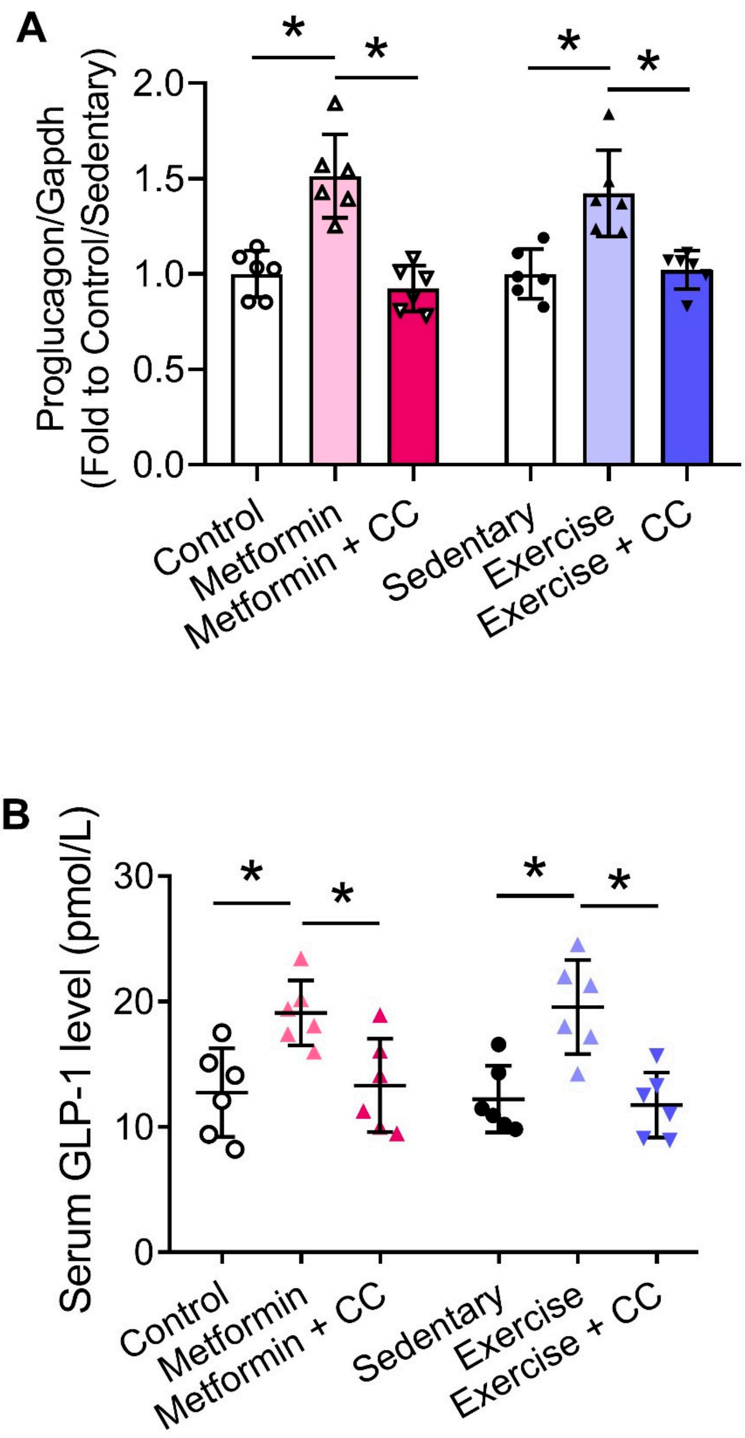


**Figure S7.** Effects of metformin treatment and exercise training on GLP-1 level. **(A)** Proglucagon mRNA level in intestines of middle-aged C57BL/6 mice after metformin treatment and exercise training in the presence and absence of compound C (CC) treatment. **(B)** Circulating GLP-1 levels in metformin-treated and exercise-trained mice. $N = 6$ per group. Data are mean ± SD. *p<0.05; Brown-Forsythe and Welch ANOVA and Dunnett T3 test.

**Part II: Supplementary table**

**Table S1.** Primer list for quantitative RT-PCR

| Genes | Forward (5′-3′) | Reverse (5′-3′) | Accession Number |
|---|---|---|---|
| E-selectin | AGTTGTGAGTTCTCCTGCGA | CACTCCATGACGCCATTCTG | NM_011345 |
| Icam1 | GTGATGCTCAGGTATCCATCCA | CACAGTTCTCAAAGCACAGCG | NM_010493 |
| Il-6 | TTCAGCCCTTGCTTGCCTC | ACACTTTTACTCCGAAGTCGGT | NM_031168 |
| Tnfα | CAGCCTCTTCTCATTCCTGC | ATGAGAGGGAGGCCATTTG | NM_013693 |
| Vcam1 | GTTCCAGCGAGGGTCTACC | AACTCTTGGCAAACATTAGGTGT | NM_011693 |
| Proglucagon | TGAGATGAGCACCATTCTGGA | TCCGCAGAGATGTTGTGAAGA | NM_008100 |
| Tert | CAGCCATACATGGGCCAGTTC | ACAGGCTGCTGCTGCTCTCA | NM_009354 |
| Gapdh | AGGTCGGTGTGAACGGATTTG | TGTAGACCATGTAGTTGAGGTCA | NM_001289726 |
| Telomere | CGGTTTGTTTGGGTTTGGGTT | GGCTTGCCTTACCCTTACCCT | N.A. |
| | TGGGTTTGGGTTTGGGTT | TACCCTTACCCTTACCCT | |
| 36B4 | ACTGGTCTAGGACCCGAGAAG | TCAATGGTGCCTCTGGAGATT | NM_007475 |
